# A new methodology for sporogony research of avian haemoproteids in laboratory-reared *Culicoides* spp., with a description of the complete sporogonic development of *Haemoproteus pastoris*

**DOI:** 10.1186/s13071-019-3832-x

**Published:** 2019-12-11

**Authors:** Dovilė Bukauskaitė, Carolina Romeiro Fernandes Chagas, Rasa Bernotienė, Rita Žiegytė, Mikas Ilgūnas, Tatjana Iezhova, Gediminas Valkiūnas

**Affiliations:** Nature Research Centre, Akademijos 2, 08412 Vilnius 21, Lithuania

**Keywords:** New methodology, *Culicoides nubeculosus*, *Haemoproteus*, Sporogony

## Abstract

**Background:**

Haemosporidian parasites of the genus *Haemoproteus* (Haemoproteidae) are widespread and cause haemoproteosis in birds and therefore, their diversity, ecology and evolutionary biology have become subjects of intensive research. However, the vectors and transmission patterns of haemoproteids as well as the epidemiology of haemoproteosis remain insufficiently investigated. Several species of *Culicoides* (Ceratopogonidae) support complete sporogony of haemoproteids belonging to the subgenus *Parahaemoproteus.* However, experimental research with these fragile insects is difficult to design in the field, particularly because their abundance markedly depends on seasonality. This is an obstacle for continuous sampling of sufficient numbers of naturally infected or experimentally exposed midges from wildlife. We developed simple methodology for accessing sporogonic development of haemoproteids in laboratory-reared *Culicoides nubeculosus*. This study aimed to describe the mosaic of methods constituting this methodology, which was applied for investigation of the sporogonic development of *Haemoproteus* (*Parahaemoproteus*) *pastoris*, a widespread parasite of the common starling *Sturnus vulgaris*.

**Methods:**

The methodology consists of the following main stages: (i) laboratory rearing of *C. nubeculosus* from the egg stage to adult insects; (ii) selection of naturally infected birds, the donors of mature gametocytes to expose biting midges; (iii) experimental exposure of insects and their laboratory maintenance; and (iv) dissection of exposed insects. Biting midges were exposed to *H. pastoris* (*cytochrome b* lineage hLAMPUR01) detected in one naturally infected common starling. Engorged insects were dissected at intervals in order to follow sporogony. Microscopic examination and PCR-based methods were used to identify the sporogonic stages and to confirm the presence of the parasite lineage in infected insects, respectively.

**Results:**

*Culicoides nubeculosus* females were successfully reared and exposed to *H. pastoris*, which completed sporogonic development 7–9 days post-infection when sporozoites were observed in the salivary glands.

**Conclusions:**

The new methodology is easy to use and non-harmful for birds, providing opportunities to access the sporogonic stages of *Parahaemoproteus* parasites, which might be used in a broad range of parasitology and genetic studies. *Culicoides nubeculosus* is an excellent experimental vector of subgenus *Parahaemoproteus* and is recommended for various experimental studies aiming investigation of sporogony of these pathogens.

## Background

Blood parasites of the genus *Haemoproteu*s (Haemosporida: Haemoproteidae) are widespread, prevalent and diverse in many terrestrial bird populations [1–3], therefore, their systematics [4], ecology [5], evolutionary biology [6–8] and genetic diversity [9] have become subjects of intensive research. For a long time, these pathogens have been considered to be relatively benign to their avian hosts and were neglected in parasitology and veterinary medicine [10]. However, recent molecular studies show that haemoproteosis may have severe and even lethal effects if this infection occurs in non-adapted avian hosts [11]. Avian haemoproteids certainly are virulent both to avian hosts and blood-sucking insects, but remain the least studied among agents of avian malaria and related haemosporidian infections. This calls for additional research aiming at a better understanding of parasite biology and disease epidemiology.

*Haemoproteus* parasites were discovered in 1890 [12], and it took over 60 years to determine that *Culicoides* biting midges are involved in the transmission of haemoproteids belonging to the subgenus *Parahaemoproteus* [13]. Experimental research with these tiny and fragile insects is challenging due to difficulties in sampling and maintaining sufficient numbers of females that support complete sporogonic development in laboratory conditions [1, 14]. This is partly because the abundance of wild *Culicoides* spp. markedly depends on seasonality, which is an obstacle for sampling sufficient numbers of exposed insects for *Haemoproteus* parasite vector research in the field [15]. During the past 65 years, 11 *Culicoides* spp. were shown to support complete sporogonic development of haemoproteids belonging to the subgenus *Parahaemoproteus* [16, 17]. However, there is still not much progress in experimental research with avian haemoproteids due to the insufficiently developed methodology for accessing the invasive stages of these parasites, sporozoites, as well as other sporogonic stages (ookinetes and oocysts). We developed such methodology using laboratory-reared biting midges *Culicoides nubeculosus*. It consists of several steps, each involving an array of simple methods, which can be applied in any laboratory. The aims of this study were to describe this methodology in detail and to use it in investigation of the sporogonic development of *Haemoproteus* (*Parahaemoproteus*) *pastoris*, a widespread parasite of the common starling *Sturnus vulgaris.*

## Methods

### Rearing of *Culicoides nubeculosus*

#### Rearing conditions

A colony of *C. nubeculosus* was established and has been maintained in the P. B. Šivickis Laboratory of Parasitology, Nature Research Centre, Vilnius, Lithuania, for 5 years. Eggs of laboratory reared *C. nubeculosus* were originally provided by the Pirbright Institute, UK. Insects were reared in a small (3 m^2^) well-aerated room with daily controlled conditions, which are air-conditioning, with a photoperiod (17:7 h day night), temperature of 25 ± 1.3 °C and relative humidity of 75 ± 5%. Humidity was maintained by permanent use of an Ultrasonic Humidifier CR 7952 (Camry, Warsaw, Poland).

#### Adult insects and eggs

**Fig. 1:**
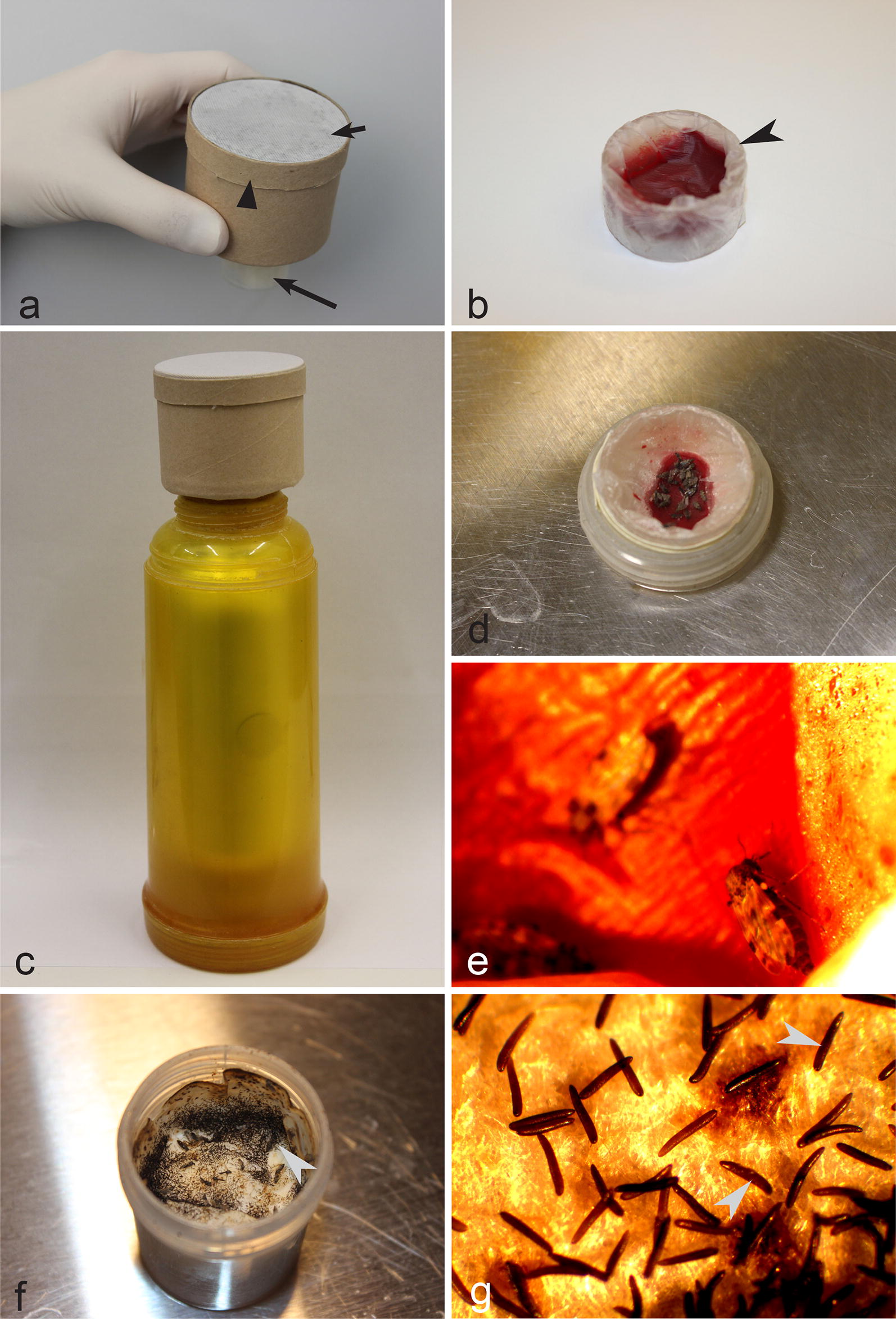
Setup for maintaining of *Culicoides nubeculosus* adult insects as well as for their blood-feeding and egg sampling. **a** Cardboard box with a colony of adult biting midges (note that each box is covered with fine-mesh bolting silk, and a plastic tube is screwed into the box bottom) short arrow, fine-mesh bolting silk and biting midges inside box; triangle arrowhead, cardboard ring, which fixes bolting silk to the insect cage; long arrow, plastic tube. **b** Small plastic container covered with parafilm and filled with blood (note that this container should be screwed into the bottom of the box (**a**) so that insects access the blood during feeding) arrowhead, small plastic container with blood covered with parafilm. **c** Thermos and a box with insects (note that insect box is placed on the top of the thermos, which is filled with warm water, resulting in heating of the plastic tube with blood). **d, e** Biting midges taking a blood meal. **f**, **g** Eggs on the cotton pad inside plastic tubes (**f**) and under a stereomicroscope at a low magnification (**g**); arrowheads, eggs which look like black spots on the filter paper (**f**) and are readily visible as elongate bodies under stereomicroscope (**g**)

Adult insects were maintained in cardboard boxes (~50 mm in diameter and height) (Xiamen Hongju Industry & Trade Co., Ltd, Xiamen, China) (Fig. 1a). One side of each box was covered with bolting silk (mesh size of ~1 mm), which was fixed by a cardboard ring, made from the box cover. A hole of ~30 mm in diameter was made in the center of the box bottom, and a plastic tube (40 mm in height and 30 mm in diameter) containing a screw thread was gently screwed into the hole. The tube can be readily screwed in or out, providing opportunities to access insects and/or their eggs. A pad of cotton moistened with tap water was placed on the bottom of the plastic tube, and a small roundish piece of filter paper was placed on the cotton pad.

**Fig. 2:**
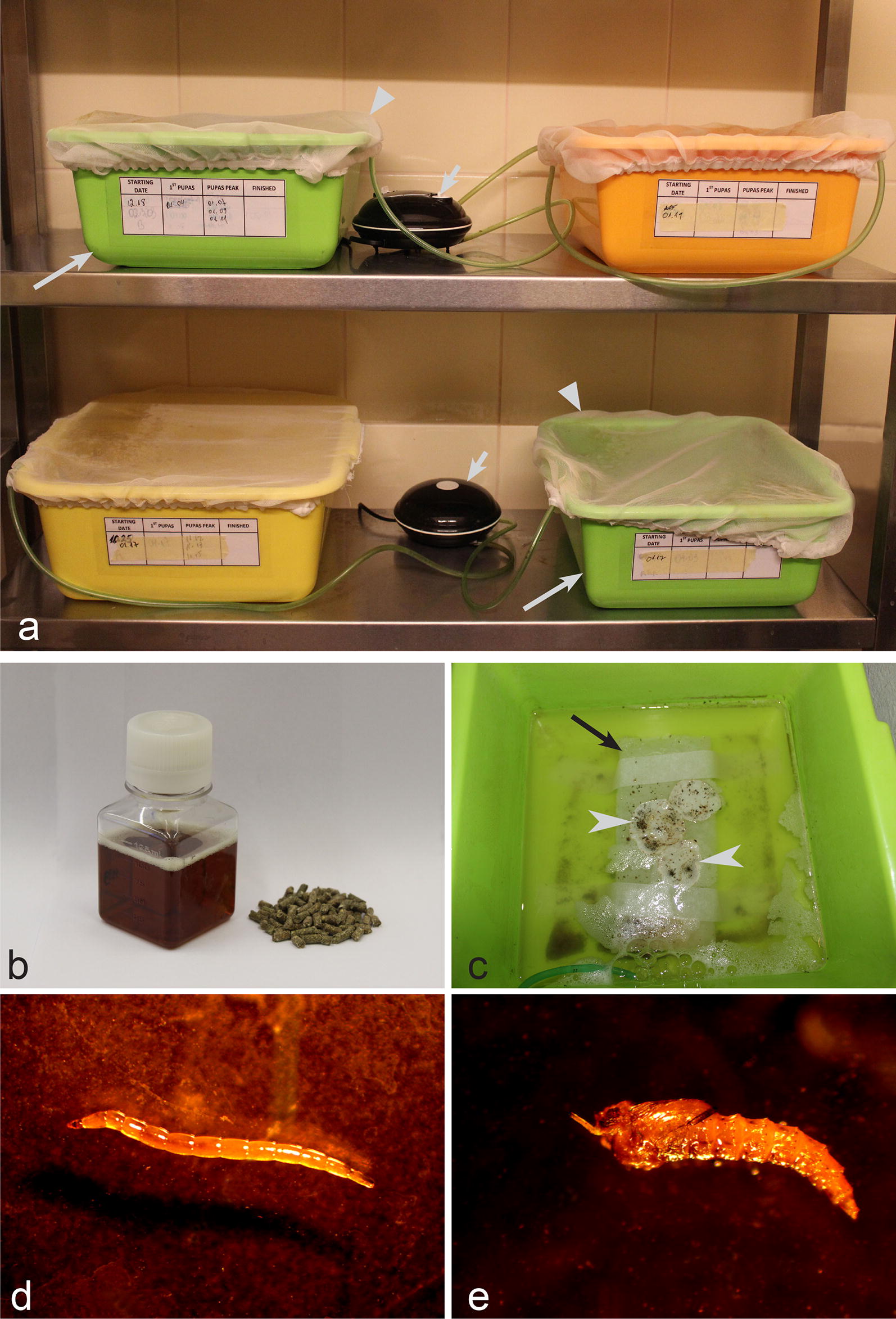
Main equipment and procedures for rearing of *Culicoides nubeculosus* from egg (**c**) to larva (**d**) and pupa stages (**e**). **a** Plastic box with larvae (note that water in each plastic box is well-aerated using air-pumps, and they are covered with a cover, which was made of fine-mesh bolting silk, preventing adults to escape if they incidentally emerge in the plastic box) long arrows, plastic boxes with water; triangle arrowheads, bolting silk cover; short arrows, air-pumps. **b** Liquid (left) and dry (right) food used to feed larvae. **c** Plastic box with eggs (note that the eggs are placed on filter paper, which is fixed on sintepon material, and the water in the plastic box is well-aerated). Long arrow, sintepon; arrowheads, eggs (which look like tiny black spots). **d** Larva (IV stage). **e** Pupa

Females of *C. nubeculosus* lay eggs on filter paper (Fig. 1f, g) located inside plastic tubes between 2 and 5 days after blood-feeding. The filter paper with eggs was removed and placed in a plastic box (30 × 30 × 10 cm) containing clean water (Fig. 2a, c). Tap water can be used, but it has to be settled for 24 h before use to allow the chlorine to evaporate. A piece of rectangular-shape of sintepon (18 × 8 × 1 cm) was maintained in each plastic box, and filter paper with eggs was placed on the sintepon (Fig. 2c). Water level should only slightly cover the top of the sintepon, on which filter paper with eggs is placed. To create optimal larvae feeding conditions, about 100 ml of water containing various microorganisms from older plastic box was also added in the new plastic box (this is not obligatory when a colony is establishing for the first time), together with 2 ml of CM0001 liquid nutrient broth (Oxoid, Basingstoke, UK) and six pellets of dry chinchilla food (UAB Akvatera, Dievogala, Lithuania). Each plastic box was equipped with permanently operating air-pumps, the same as commonly used in small aquaria (Aquael, Warsaw, Poland, or similar ones). Additionally, the plastic boxes were covered with bolting silk (Fig. 2a), which prevented the escape of insects in cases of incidental emergence of adults before pupae collection.

#### Larvae maintenance

Larvae were fed three times per week with 1 ml CM001 liquid nutrient broth (Oxoid), and three granules of dry chinchilla food (UAB Akvatera, Dievogala, Lithuania), per plastic box (Fig. 2b). During growth, *C. nubeculosus* larvae moult four times [18].

#### Pupae

Larvae (Fig. 2d) become pupae (Fig. 2e) between two and three weeks after emergence from eggs. Fourth-instar larvae (Fig. 2d) move to the sintepon and moult into pupae (Fig. 2e). Pupae were collected every day and placed on the top of filter paper, which was then placed in plastic tubes fixed to carboard boxes, as described for adult insects above. The tubes with pupae were screwed into the bottom of the insect box (Fig. 1a) where adult insects emerged. Approximately 600–800 pupae were placed in each cardboard box for colony maintenance.

#### Adult insects

**Fig. 3:**
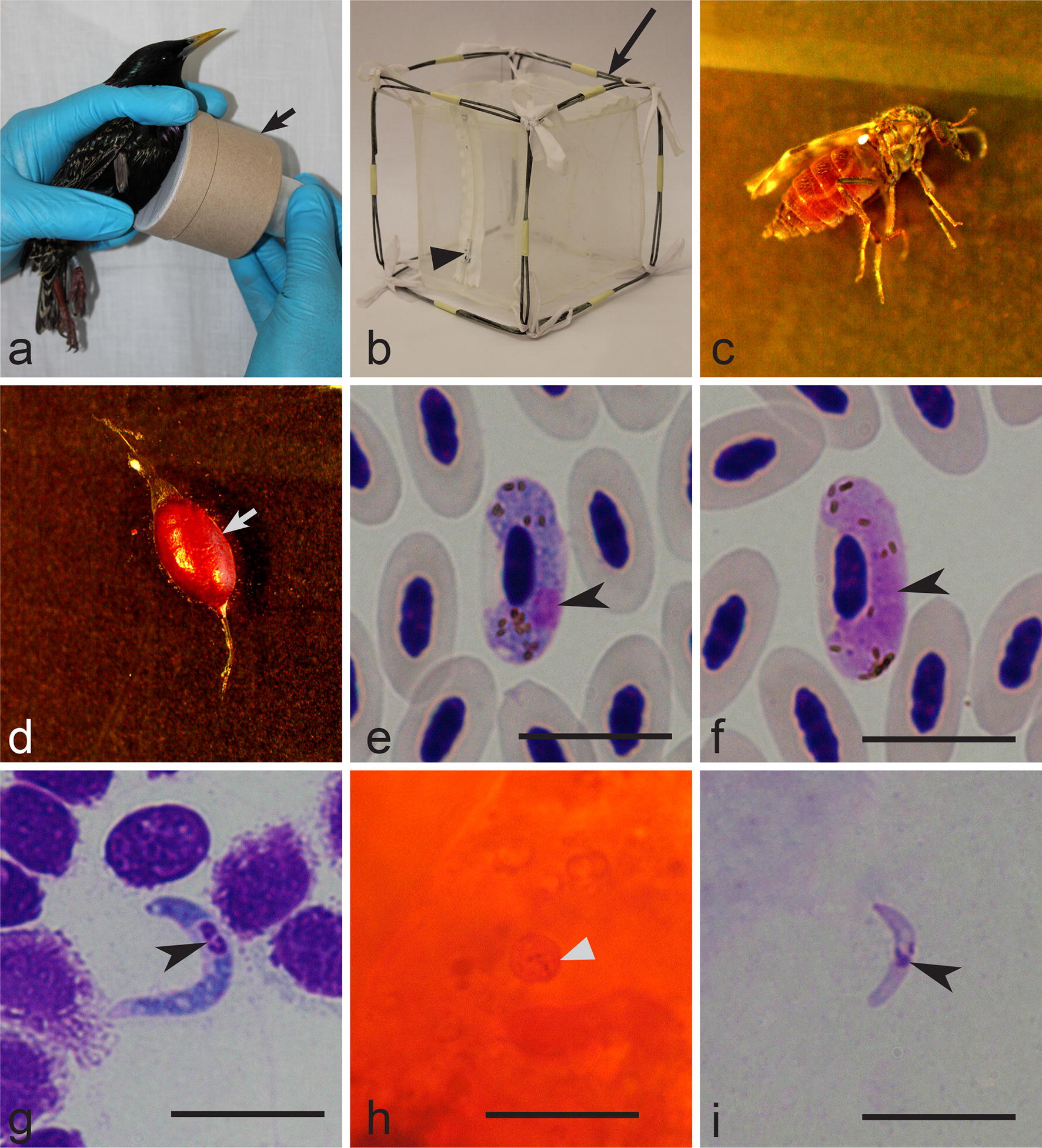
Main procedures of exposure of *Culicoides nubeculosus* colony to *Haemoproteus pastoris* (lineage hLAMPUR01) infection by allowing the insects to take blood meal on naturally infected common starling *Sturnus vulgaris* (**a**, **b**). Note that exposure was done by gentle touching of a cardboard box (arrow) with biting midges to the pectoral muscle of the infected bird (**a**) and then engorged insects are released into a larger insect cage (**b**) (note that a zipper is sewed into one side of the cage) long arrow, insect cage; triangle arrowhead, zipper. Exposed females (**c**) were maintained in insect cage made of bolting silk (**b**) and their midguts were dissected (**d**) (arrow, dissected midgut full with blood). Mature gametocytes (**e**, **f**) were present in the peripheral blood of the infected starling (arrowheads, parasite nuclei), and the sporogonic stages (**g–i**) developed in exposed female biting midges: macrogametocyte (**e**); microgametocyte (**f**); ookinete (**g**) (arrowhead, parasite nuclei); oocyst (**h**) (arrowhead, oocyst); sporozoite (**i**) (arrowhead, parasite nuclei). Giemsa (**e**–**g**, **i**) and mercurochrome (**h**) stained preparations. *Scale*-*bars*: 10 µm

Adults emerge approximately 1–2 days after the collection of pupae (Fig. 3c). They were fed daily by placing a cotton pad moistened with 10% sugar on the top of the insect box. Females are ready to take a blood meal after 3–4 days post-emergence and then lay eggs 2–5 days after blood-feeding. Females were particularly active blood-feeders between 4–7 days post-emergence, and this is the most productive time period for the experimental infection.

#### Blood-feeding of females

A small plastic container (15 × 25 mm) (Fig. 1b) was filled with blood (dog, horse or other bird blood can be used). The blood should contain anticoagulant; sodium citrate was used in the proportion of 1 part of the anticoagulant and 4 parts of blood (sodium heparin in the same proportion also can be used). This container was then covered with parafilm, which should be gently stretched around the container forming a thin layer and attached to the surface of the blood (Fig. 1b). The container with blood was slightly warmed by placing it on the surface of warm water (~50 °C) for 5 min; the container with blood was then fixed into a plastic cylinder (the same diameter as plastic tubes; see Fig. 1d), and screwed into the bottom of the insect box (Fig. 1a) containing adult biting midges. A thermos with warm water (approximately 40 °C) was used to keep the blood warm (Fig. 1c). Females immediately start blood-feeding (Fig. 1d, e), and fly off when fully engorged. After 4–5 days they lay eggs, which were used for further maintenance of the colony. The ‘egg to egg’ cycle occurred in approximately a 4–5-week period.

### Selection of naturally infected birds, the donors of mature gametocytes to expose *C. nubeculosus*

Parasite-infected birds were selected at Ventės Ragas Ornithological Station, Lithuania in May 2015. Birds were caught with mist nets, zigzag traps and a big Rybachy-type trap. In all, five common starlings (*Sturnus vulgaris*) were captured and blood was collected from the brachial vein. Approximately 30 µl of blood was collected with a heparinized microcapillary tube and stored in non-lysis SET buffer (0.05 M Tris, 0.15 M NaCl, 0.5 M EDTA, pH 8.0) for molecular analysis. The samples were kept at ambient temperature in the field and later at − 20 °C in the laboratory. Three small drops of freshly collected blood were used to make three blood films, which were rapidly dried using a battery-operated fan and fixed with absolute methanol. Two blood films were stained with 10% Giemsa solution, as described by Valkiūnas [14]. They were used for detailed microscopic examination in the laboratory. One blood film was used for rapid parasite diagnostic (see description below).

While being checked for haemosporidian infections, the captured birds were maintained in standard cloth bags. One blood film from each bird was stained rapidly with 30% Giemsa solution for 15 min and examined under a high magnification (×1000) microscope within 5 min after staining. The main aims of this rapid examination were to select an infected bird with gametocytaemia of approximately 0.1–1.0%, and also to exclude possible co-infections with other *Haemoproteus* parasites in the donor bird. Uninfected birds and birds with too low (< 0.1%) or too high (> 1.0%) parasitaemia were released within 1 h after their capture; these birds are not suitable for use in experimental research because high *Haemoproteus* parasite parasitaemia kills insects, and in cases of too low gametocytaemia sporogonic stages are difficult to visualize in insect preparations [14, 19]. One bird with high parasitaemia of mature gametocytes (1.9%) was used for initiation of an *in vitro* sexual process and ookinete development (see description below).

One common starling with 0.3% parasitaemia of *H. pastoris* was selected as a donor of mature gametocytes for experimental exposure of biting midges. Parasite species identification was achieved using morphological characters of gametocytes [14]. This infected starling (donor of gametocytes) was used to expose biting midges immediately after determining gametocytaemia, which happened approximately 1 h after the bird was caught.

In the laboratory, blood films were examined additionally to double check the results of the fast field observations; blood films of experimental birds were examined for 15–20 min at a low magnification (×400), and then at least 100 fields were studied at a high magnification (×1000). The intensity of gametocytaemia was determined as a percentage by actual counting of the number of mature gametocytes per 1000 red blood cells. All vectors preparations were examined at a high (×1000) magnification. An Olympus BX-43 light microscope equipped with an Olympus SZX2-FOF digital camera and imaging software Qcapture Pro 6.0, Image Pro plius (Olympus, Tokyo, Japan) were used. Statistical analyses were carried out using R studio version 3.4.3. [20]. Representative preparations of blood stages (49148NS) and vector stages (ookinetes 49149NS, oocysts 49150NS and sporozoites 49151NS) were deposited in the Nature Research Centre, Vilnius, Lithuania.

### Experimental exposure of *C. nubeculosus* and their laboratory maintenance

One common starling naturally infected with *H. pastoris* was used to infect the biting midges. Insect boxes were transported to the field laboratory and maintained at similar conditions as the original colony. An insect box containing approximately 70–80 insects (usually, 50% of them are females) was gently touched to the feather-free area on the pectoral muscles of an infected bird (Fig. 3a). Female midges willingly took a blood meal through the bolting silk, and majority of them were fully engorged after approximately 30 min. The donor bird was released immediately after insect exposure, and remained in good condition.

The experimental insects were transferred from the insect box (Fig. 3a) into a cage made of bolting silk and fixed to a wire frame (12 × 12 × 12 cm) (Fig. 3b). A zipper was sewn into one of its sides, which allows the researcher to reach inside and perform the necessary manipulations. Males and unfed females were removed. About 20–40 engorged females were usually present inside each insect cage; they were kept in a room with approximately the same conditions as the main colony; temperature was maintained around 22–23 °C. Cotton pads moistened with 10% solution of sugar were placed on the top of each cage in order to feed insects daily.

### Dissection of exposed insects

Infected females were dissected and preparations of sporogonic stages (ookinetes, oocysts and sporozoites) were prepared as described by Valkiūnas [14]. Biting midges were caught with an insect aspirator. Before dissection, insects were anesthetized in a tube covered with cotton-wool pads moistened with 96% ethanol.

Ookinete preparations were prepared 3–6 h post-infection. Midgut (Fig. 3d) was gently extracted, crushed on a glass slide, and a thin film was prepared. These preparations were fixed with absolute methanol and stained with 10% Giemsa solution for 1 h.

Oocyst preparations were made between 4 and 7 days post-infection (dpi). It is important not to damage the midguts during the isolation. The isolated midguts were placed on microscope glass slides. A drop of 2% mercurochrome solution was added on each midgut, which was then covered with a coverslip, and oocysts were visualised in these temporary preparations. If present, photographs of oocysts were made. Permanent oocyst preparations were made from best fresh preparations following Valkiūnas [14]. Briefly, midguts were fixed in 10% neutral (pH = 7.0) formalin solution for 24 h. Then, formalin was removed by placing the preparations in 70% ethanol for 6 h. Later, the preparations were washed with distilled water, stained with Ehrlich’s haematoxylin for 10 min, placed in water with a pinch of sodium bicarbonate and differentiated with acid ethanol for 5 min, and placed again in water with sodium bicarbonate. Then, the preparations were dehydrated with 70% and then with 96% ethanol. A drop of each, clove oil and xylene, were used to clear the preparation. Finally, a drop of Canada balsam was placed on the midguts, which were covered with coverslips and air-dried for 2–3 weeks.

Sporozoite preparations were made between 5 and 7 dpi. Salivary glands were isolated and gently crushed on glass slides. Small thin smears (about 5 mm^2^) were prepared, fixed in methanol and stained with 4% of Giemsa solution for 1 h. Due to the tiny size of the glands, remnants of insect thorax tissue usually remain in such preparations, but this does not prevent sporozoite observation.

After each insect dissection, residual parts of their bodies were fixed in 96% ethanol and used for PCR-based analysis in order to confirm the presence of corresponding parasite lineages in vectors. Dissection needles were flame-disinfected to prevent contamination during the dissections.

### *In vitro* development of *H. pastoris*

One individual common starling with a high parasitaemia of mature gametocytes (1.9%) was used as the donor of parasites for *in vitro* examination of sexual process and ookinete development of *H. pastoris*. A protocol by Valkiūnas et al. [21] was used for initiation of the *in vitro* development. Approximately 200 µl of blood was taken from the brachial vein using heparinized microcapillary tubes, immediately placed in a 2 ml Eppendorf tube and mixed with a 3.7% solution of sodium citrate in a ratio of 1 part of solution to 4 parts of blood. To prevent the drying of the solution with blood, each Eppendorf tube was placed in a 50 ml polypropylene container with a sheet of filter paper moistened in water on its bottom. A cap was screwed on the container tube. The experiment was performed at 22 °C. Smears were prepared from the blood-citrate mixture at set intervals of time after exposure to air (1, 3, 5, 10, 15, 30, 45, 60 min, and 2, 3, 4, 6, 8, 10, 12, 16, 24 h). Smears were air-dried, fixed in methanol, stained with 10% Giemsa solution for 1 h, and examined under a microscope at a high magnification (×1000), as blood films (see description above).

### PCR, sequencing and phylogenetic analysis

Total DNA was extracted from all samples using the standard ammonium acetate extraction method [22]. A nested PCR protocol was used to amplify a 479-bp fragment of the *cytochrome b* gene (*cytb*) [23, 24]. The primers HaemNFI and HaemNR3 were used to amplify fragments of *Haemoproteus*, *Plasmodium* and *Leucocytozoon* parasites. The primers HaemF and HaemR2 were applied for the second PCR, which amplifies DNA of *Haemoproteus* and *Plasmodium* parasites. The success of the performed PCR was evaluated by running 1.5 µl of PCR product on a 2% agarose gel. One negative control (nuclease-free water) and one positive control (a *Haemoproteus* sp.-infected sample, which was selected as positive by microscopic examination) were used every 7 samples to control for possible false amplifications.

**Fig. 4:**
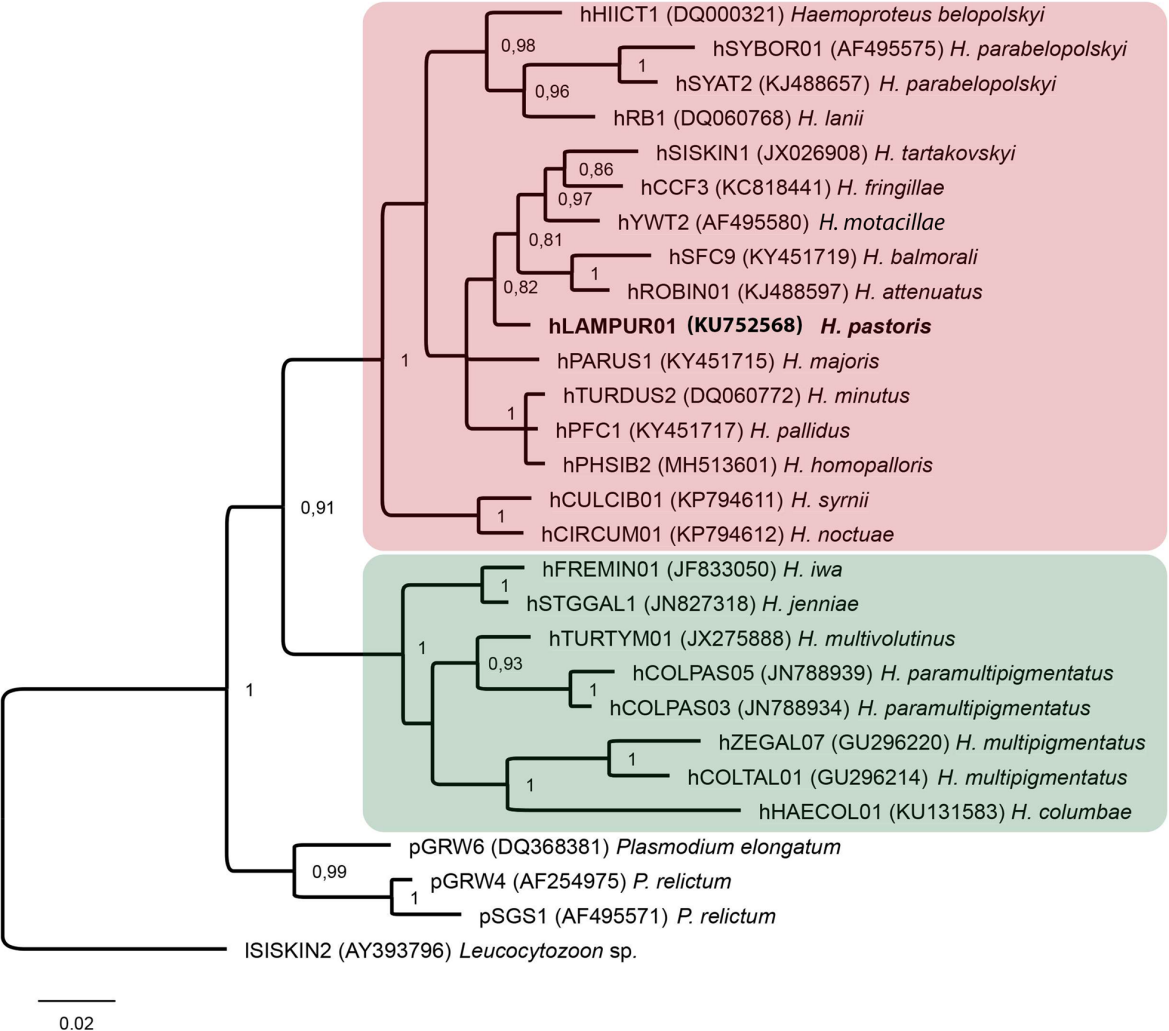
Bayesian phylogeny of the mitochondrial *cytochrome b* lineages (479 bp) of avian *Haemoproteus* parasites, which are transmitted by biting midges (Ceratopogonidae) (pink box) and louse flies (Hippoboscidae) (green box). The tree was rooted with *Leucocytozoon* sp. (lineage lSISKIN2), and is drawn to scale based on inferred substitutions per site. Codes of the lineages are given according to the MalAvi database [27]; GenBank accession numbers of the sequences are provided in parentheses. The parasite lineage used in this study is indicated in bold

Fragments of DNA from the PCR-positive amplifications were sequenced. Big Dye Terminator V3.1 Cycle Sequencing Kit and ABI PRISM™ 3100 capillary sequencing robot (Applied Biosystems, Foster City, California) were used for sequencing. Sequences were aligned in BioEdit software [25]. Double-base calling (double peaks) in electropherograms was considered as an indication of mixed infection [26]. The NCBI nucleotide BLAST (Basic Local Alignment Search Tool) running the mega blast algorithm (http://blast.ncbi.nlm.nih.gov/Blast.cgi) was used to identify the lineages of haemosporidian parasites. Bayesian phylogenetic analysis was carried out using our samples and sequences from GenBank. All sequences were double-checked in the MalAvi database [27]. A *Leucocytozoon* sp. sequence was used as the outgroup. Codes of the lineages and the GenBank accession numbers for the sequences are provided in the phylogenetic tree (Fig. 4). Bayesian phylogenetic tree (Fig. 4) was constructed implementing MrBayes version 3.1 [28] software. The General Time Reversible Model (GTR) was selected as the best-fitting model by the software MrModeltest 2.2 [29]. The analysis was run for a total of 5 million generations with a sampling frequency of every 100 generations. Before the construction of the consensus tree, 25% of the initial trees were discarded as ‘burn in’ period. The tree was visualized using the software FigTree v1.4.3 (http://tree.bio.ed.ac.uk/software/figtree/).

## Results

Both microscopic examination and PCR-based testing showed the presence of single *Haemoproteus* infections in the parasite donor bird. *Haemoproteus pastoris* (*cytb* lineage hLAMPUR01) was identified (Figs. 3e, f, 4). Polymerase chain reaction and sequencing confirmed the presence of this parasite lineage in experimentally infected insects.

### Sporogonic development of *H. pastoris in vivo*

In all, 14 females were engorged after experimental exposure; 2, 6 and 6 females were dissected for detection of ookinetes, oocysts and sporozoites, respectively. *Haemoproteus pastoris* completed sporogony in experimentally infected *C. nubeculosus*. Mature ookinetes of *H. pastoris* were observed between 3 and 6 h post-infection (Fig. 3g, Table 1). They are elongated worm-like bodies, with slightly off-centre located nuclei and a markedly vacuolated cytoplasm. Young oocysts were first observed 2 dpi, and they were present until 5 dpi. Pigment granules were visible in some oocysts (Fig. 3h). Sporozoites were observed in salivary gland preparations between 7–9 dpi, indicating that this biting midge supports complete sporogony and likely is a natural vector of *H. pastoris*. Sporozoites (Fig. 3i, Table 1) were elongated, with centrally or slightly off-centre located nuclei. Because a donor bird with low gametocytaemia was used for insect exposure, the number of reported sporogonic stages was small (Table 1) and these stages were difficult to find in preparations.

**Table 1:** Morphometric parameters of ookinetes and sporozoites of *Haemoproteus pastoris* (lineage hLAMPUR01) in experimentally infected biting midges *Culicoides nubeculosus*

Feature	Ookinetes (n = 21)	Sporozoites (n = 13)
Length	12.8–16.3 (14.5 ± 0.9)	6.2–11.3 (8.3 ± 1.5)
Width	1.8–3.1 (2.2 ± 0.4)	1–1.8 (1.2 ± 0.2)
Area	19.1–33.8 (25.8 ± 4.6)	6.3–12.8 (9.3 ± 2.0)

*Notes*: Measurements are given in micrometres. Minimum and maximum values are provided, followed by the arithmetic mean and standard deviation in parentheses

### *In vitro* development of *H. pastoris*

**Fig. 5:**
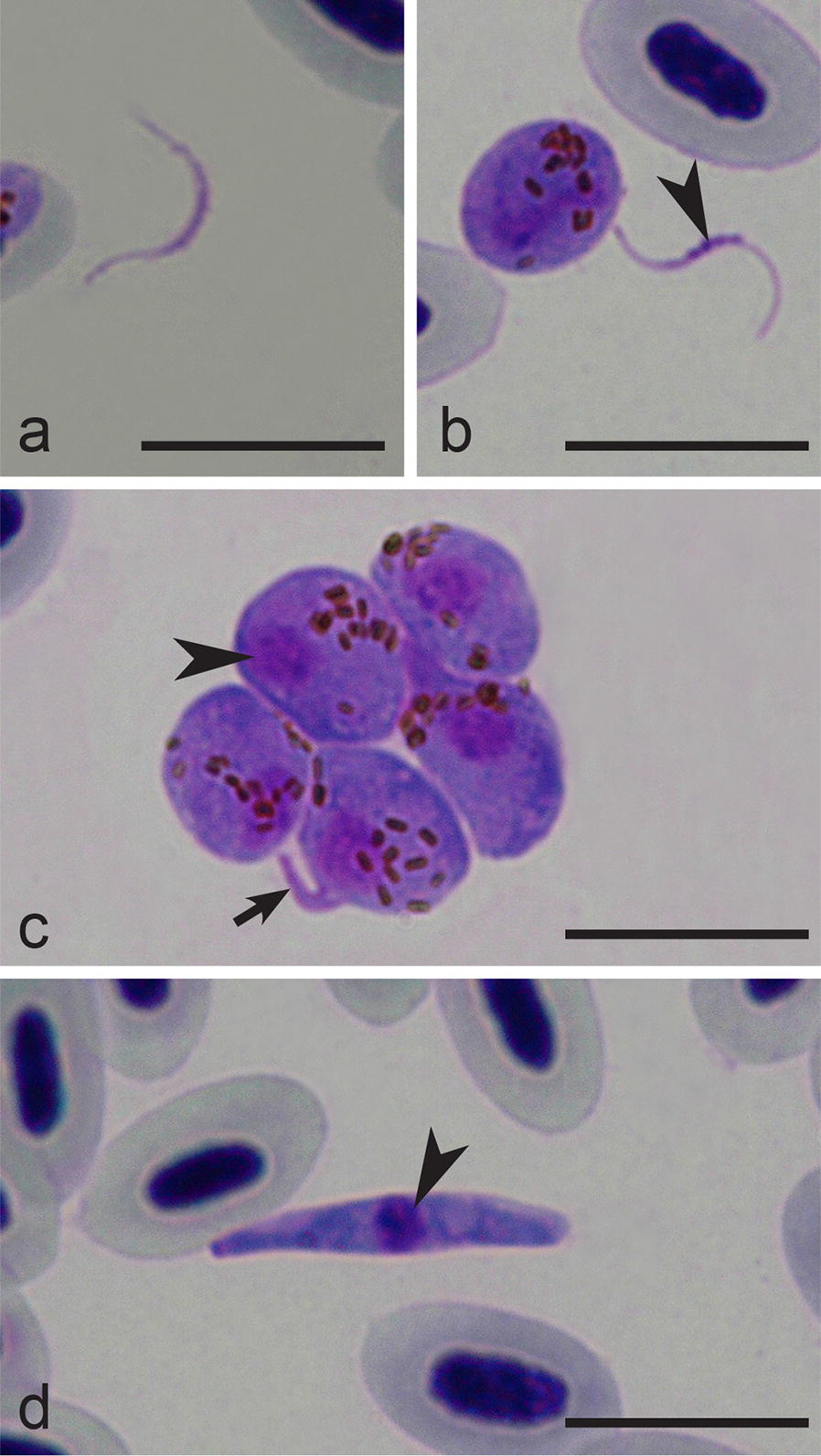
*In vitro* stages (**a**–**d**) of *Haemoproteus pastoris* (hLAMPUR01). **a** Microgamete. **b** Macrogamete (arrowhead, parasite nuclei). **c** Group of zygotes (note the appearance of a small outgrowth (short arrow) in one parasite indicating initial stage of ookinete development; arrowhead, parasite nuclei). **d** Mature ookinete (arrowhead, parasite nuclei). Giemsa-stained thin films. *Scale*-*bars*: 10 µm

Exflagellation, formation of macro- and microgametes, fertilization and development of ookinetes was readily induced *in vitro*. Numerous microgametes (Fig. 5a), macrogametes and fertilization events (Fig. 5b), zygotes (Fig. 5c) and fully grown ookinetes (Fig. 5d) were observed. Numerous exflagellating microgametocytes and microgametes were detected 5–10 min after exposure to air. At the same time, first fertilizations of macrogametes were observed (Fig. 5b). Developing ookinetes contain outgrowths, which correspond to apical ends of the parasites (Fig. 5c). Ookinetes developed rapidly, and were first observed 2 h post-exposure; the majority had features of mature ookinetes 6 h post-exposure (Fig. 5d). Ookinetes *in vitro* and *in vivo* were similar; the majority of fully-grown parasites lacked pigment granules, which were eliminated from ookinetes during the development of the parasite residual bodies.

### Phylogenetic analysis

In accordance with the complete sporogonic development in *C. nubeculosus*, the phylogenetic analysis placed the lineage hLAMPUR01 in a well-supported clade of haemoproteid species transmitted by *Culicoides* biting midges and belonging to the subgenus *Parahaemoproteus* (Fig. 4). Haemoproteids belonging to the subgenus *Haemoproteus*, transmitted by hippoboscid louse flies, appeared in a well-supported sister clade.

## Discussion

A number of molecular studies have reported the presence of *Haemoproteus* spp. genetic lineages in *Culicoides* spp. biting midges [30–33], without convincing evidence that the parasites complete sporogonic development and produce invasive sporozoites, an essential stage of transmission. These studies are important because they indicate natural links between avian hosts and blood-sucking insects in the wild, but are insufficient to prove that insects can act as vectors. Demonstration of sporozoites in salivary glands of insects is an essential next stage in vector research [34].

*Culicoides nubeculosus* was used for *Haemoproteus* spp. vector research by Miltgen et al. [35] for the first time in 1981. It was shown that this biting midge supports sporogonic development of *Haemoproteus handai*, a virulent parasite of parrots [36]. According to previous studies [17, 37, 38], nine widespread species of the subgenus *Parahaemoproteus* complete sporogony in *C. nubeculosus*; ookinetes and oocysts were reported in the midgut and sporozoites developed and reached the salivary glands, indicating that this insect is a competent vector and might transmit these infections naturally. It is important to note that some of these haemoproteid species (*H. handai*) are parasites of tropical origin, with no transmission in temperate regions [14]. In other words, *C. nubeculosus* readily supports sporogony of tropical parasites, which migrating birds bring to the Palaearctic breeding grounds. Because the potential vectors (species of *Culicoides*) are available in the Palaearctic ([16, 17], the present study) and the avian haemoproteids might be virulent and even lethal in non-adapted avian hosts [11], epidemiological conditions might be created for spreading of pathogens with tropical origin in countries with temperate climates, if the ecological situation changes. This calls for more attention in bird management and veterinary medicine regarding haemoproteosis epidemiology.

Methods of laboratory maintenance of *C. nubeculosus* developed by Boorman [39] were modified in this study in regard to blood-feeding of insects and experimental exposure to *Haemoproteus* infections (Fig. 1b–e). Methods of experimental exposure of females were developed and successfully used in our laboratory [17, 37, 38], but remained undescribed in detail. The protocols, which were applied in the present study, are relatively simple and can be developed in any laboratory. Importantly, they are not harmful to birds. The described array of methods is recommended for application in vector research of avian haemoproteids. Importantly, the described methodology provides opportunities to access *in vivo* the sporogonic stages (oocysts, ookinetes and sporozoites), which are difficult to access in wildlife, for various studies aiming investigation of issues related to sporogony and genetics of these parasites. For example, laser microdissection techniques might be used to access single cells of the parasites at each stage of sporogony [40].

The present study identified *C. nubeculosus* as a vector of *H. pastoris*. This blood parasite was added to the group of avian haemoproteids which complete the sporogonic development in *Culicoides* species [17]. *Haemoproteus pastoris* is prevalent in common starling [14], an invasive bird species [42]. These birds are spreading worldwide and might transport this infection to new ecosystems during dispersal. Haemoproteids of common starlings deserve more attention in regard to transport of infections by migrating birds globally and can be used as convenient model organisms for biogeography research. The common starling and *C. nubeculosus* co-exist in woodlands in Eurasia [42, 43].

It is important to note that phylogenetic analysis placed the *cytb* lineage of *H. pastoris* into a well-supported clade of *Parahaemoproteus* parasites, which are all transmitted by *Culicoides* biting midges; louse fly-transmitted parasites of the subgenus *Haemoproteus* appeared in a sister clade (Fig. 4). This finding further strengthens previous conclusions that phylogenies based on partial *cytb* genes readily distinguish major groups of haemoproteid vectors [17]. The finding is thus helpful in planning vector research of bird haemoproteids. Basically, *cytb* sequences of many bird haemoproteids are readily accessible and phylogenetic analyses are easy to carry out. Availability of such data provide clear directions in vector research of bird parasites, e.g. for not yet studied vectors for which the group of possible dipteran vectors (Ceratopogonidae or Hippoboscidae) is unknown. This is a particularly sensitive issue because the vectors of haemoproteids of common birds belonging to many orders, e.g. Falconiformes, Accipitriformes, Charadriiformes, Ciconiiformes, Coraciiformes, Cuculiformes, Piciformes, remain unidentified. According to available data, *Culicoides* biting midges support the sporogonic development of haemoproteids parasitizing birds of the Passeriformes, Anseriformes, Galliformes, Piciformes, Psittaciformes, Strigiformes and Galliformes [1, 14, 16, 17]. Thus far, louse flies have been reported to support the sporogonic development of haemoproteids parasitizing birds belonging only to Columbiformes, Charadriiformes and Pelecaniformes [44]. The factors inhibiting the sporogonic development of *Parahaemoproteus* and *Haemoproteus* species in louse flies and biting midges, respectively, remain unclear. Further experimental studies might provide insights into vector biology of avian haemoproteid parasites, which are of particular interest due to their cosmopolitan distribution and severe diseases caused in non-adapted avian hosts [11, 45].

The rate of ookinete formation varies from 1–4 h in *Haemoproteus minutus* to 12 h and even more in *Haemoproteus tartakovskyi* and many other haemoproteid species at similar temperature conditions [14]. In other words, ookinetes of *H. minutus* disappear from vector midguts soon after infection and can hardly be detected 4 h post-infection, but are numerous in the case of *H. tartakovskyi* and many other haemoproteids at the same time interval [16]. Information about the rate of ookinete formation is valuable to plan *in vivo* vector research, particularly when detection of ookinetes is aimed. Our study shows that *H. pastoris* readily develops in *in vitro* conditions, with a relatively fast (2–6 h post-infection) development of mature ookinetes.

*Haemoproteus pastoris* belongs to a group of avian haemoproteids, which produce ookinetes displaying outgrowths at the initial stages of their development (Fig. 5c), as is the case in *H. attenuatus*, *H. balmorali*, *H. belopolskyi*, *H. fringillae*, *H. hirundinis*, *H. lanii*, *H. motacillae*, *H. parabelopolskyi* and *H. tartakovskyi*, but not in *H. minutus* and *Haemoproteus pallidus* [14, 46]. The biological meaning of this transformation remains unclear. It is worth mentioning that zygotes of *H. pastoris* tend to group during development *in vitro*, with numerous zygotes reported in clumps containing from few to tens of organisms (Fig. 5c). This provides opportunities to dissect such parasite clumps for genetic studies using laser microdissection techniques [40, 41] and obtain clean parasite DNA material, avoiding contamination with avian host DNA. The latter issue remains an obstacle in genomic research of haemosporidian parasites inhabiting nucleated red blood cells of reptiles and birds [9, 47, 48].

## Conclusions

The methodology for laboratory rearing of *C. nubeculosus* and its experimental exposure to avian *Haemoproteus* infections was developed, tested and described in this study. *Culicoides nubeculosus* is an excellent experimental vector of avian haemoproteids belonging to the subgenus *Parahaemoproteus* due to easy maintaining in laboratory. Additionally, this biting midge is readily susceptible to many avian haemoproteid species and is recommended for experimental sporogony research and accessing of *in vivo* sporogonic stages of *Haemoproteus* blood parasites. The described methodology constitutes of simple methods, which can be developed in any laboratory and thus provides opportunities for extending experimental research with avian haemoproteids broadly. *Haemoproteus pastoris* completes sporogonic development in *C. nubeculosus*, which likely is a natural vector of this widespread parasite. Additionally, the exflagellation and ookinete development of *H. pastoris* and many other avian haemoproteids readily occur *in vitro*, providing opportunities for sampling gametes, zygotes and ookinetes of these pathogens for genetic studies. It is highlighted that data on the rate of *in vitro* development of haemoproteid ookinetes are useful for designing *in vivo* sporogony studies, worth accumulating for many these parasite species.

## Data Availability

The data that support findings of this study are included within the article. Representative preparations of vector stages were deposited in Nature Research Centre, Vilnius, Lithuania, under the accession numbers 49148NS (blood stages), 49149NS (ookinetes), 49150NS (oocysts) and 49151NS (sporozoites).

## Abbreviations

DNA: deoxyribonucleic acid
*cytb*: mitochondrial *cytochrome b* gene
PCR: polymerase chain reaction
dpi: days post-infection
